# The effects of motivational interviewing on patients with comorbid substance use admitted to a psychiatric emergency unit - a randomised controlled trial with two year follow-up

**DOI:** 10.1186/1471-244X-13-93

**Published:** 2013-03-21

**Authors:** Gunnhild Bagøien, Johan Håkon Bjørngaard, Christine Østensen, Solveig Klæbo Reitan, Pål Romundstad, Gunnar Morken

**Affiliations:** 1 Østmarka Department of Psychiatry, St. Olav University Hospital, Trondheim, Norway; 2 Department of Neuroscience, Norwegian University of Science and Technology, Trondheim, Norway; 3 Department of Public Health and General Practice, Norwegian University of Science and Technology, Trondheim, Norway; 4 Forensic Department and Research Centre Bröset, St. Olav University Hospital Trondheim, Norway; 5 Department of Research and Development, Psychiatry, St. Olav University Hospital, P O Box 3008 Lade, Trondheim, NO-7441, Norway

**Keywords:** Motivational interview, Psychiatric emergency services, Comorbidity, Substance abuse, Hospitalisation

## Abstract

**Background:**

The prevalence of substance use in people acutely admitted to in-patient psychiatric wards is high and the patients` duration of stay is limited. Motivational interviewing is a method with evidence based effect in short interventions. The aims of the present study were to compare the effects of 2 sessions of motivational interviewing and treatment as usual (intervention group) with treatment as usual only (control group) on adult patients with comorbid substance use admitted to a psychiatric in-patient emergency unit.

**Methods:**

This was an open randomised controlled trial including 135 patients where substance use influenced the admittance. After admission and assessments, the patients were allocated to the intervention group (n = 67) or the control group (n = 68). The primary outcome was self-reported days per month of substance use during the last 3 months at 3, 6, 12 and 24 months after inclusion. Data was analysed with a multilevel linear repeated measures regression model.

**Results:**

Both groups reduced substance use during the first 12 months with no substantial difference between the 2 groups. At 2 year follow-up, the control group had increased their substance use with 2.4 days (95% confidence interval (CI) –1.5 to 6.3), whereas the intervention group had reduced their monthly substance use with 4.9 days (95% CI 1.2 to 8.6) compared to baseline. The 2 year net difference was 7.3 days of substance use per month (95% CI 1.9 to 12.6, p < 0.01) in favour of the intervention group.

**Conclusions:**

The present study suggests that 2 sessions of motivational interviewing to patients with comorbid substance use admitted to a psychiatric emergency unit reduce substance use frequency substantially at 2 year follow-up.

**Trial registration:**

ClinicalTrials.gov Identifier: NCT00184223

## Background

Patients with psychiatric disorders have higher rates of substance use disorders than the general population [1]. Among patients acutely admitted to psychiatric in-patient wards the use of alcohol and illicit drugs is high [2]. Proportions of substance use disorders in these patients range from 32 to 56% in previous studies [3,4]. In some studies on patients with psychiatric disorders, alcohol and drug use are found to be associated with more severe symptoms, poorer treatment adherence [5-7] and a higher risk of relapses and admissions [8]. Patients with substance use have a short length of stay in psychiatric emergency units [9,10], giving limited time for treatment interventions. Suitable treatments to prevent recurrence of problematic substance use after detoxification and discharge are therefore needed.

Motivational interviewing developed by William R. Miller and Stephen Rollnick [11], is a directive, client-centered method for enhancing intrinsic motivation to change by exploring and resolving ambivalence, increasing the patients` own view of the importance of change and emphasizing the patients` freedom of choice. The method has been applied in a wide variety of settings and includes different health behaviours [12]. A recent Cochrane review concludes that motivational interviewing has a significant effect on substance use when compared to no treatment, but no difference compared to treatment as usual. The effects tend to be seen early but diminish during one year follow-up [13]. Studies with a follow-up longer than 12 months are sparse [14,15]. Interestingly, a study has shown that psychotherapy inducing intrinsic change and insight actually has been shown to have clinical effect after two years [16].

The documentation of the effect of motivational interviewing on patients with combined substance use and psychiatric disorders is inconclusive [17-20]. Motivational interviewing is often integrated in combined psychosocial interventions [21] and studies include mainly patients with severe mental illnesses (e.g. psychoses) [22,23] ignoring the major group with combined substance use and affective disorders.

Randomised controlled trials with long term follow-up on manual-guided psychosocial interventions to patients with substance use and co-occurring depression, anxiety or psychotic disorders are few [24,25].

The aims of the present open randomised controlled trial were to investigate the effects of individually administered motivational interviewing on patients with comorbid substance use admitted to a psychiatric emergency unit. We hypothesized that the patients receiving both motivational interviewing and treatment as usual would reduce the frequency of substance use after discharge more than the patients receiving treatment as usual only.

## Methods

### Study design and randomisation

This study was a stratified randomised controlled open clinical trial, where consecutive patients were allocated either to the combination of motivational interviewing and treatment as usual (intervention group), or treatment as usual only (control group).

### Ethics

The study was approved by the Regional Committee for Medical Research Ethics, (identification number 110–04) Middle Norway and Norwegian Social Science Data Services (NSD).

### Participants

At the time of the inclusion, St. Olav University Hospital, Østmarka Department of Psychiatry was the only emergency psychiatric in-patient service for adult patients from 18 years old in a catchment area of about 140 000 inhabitants from a geographical area covering 50% of the population of the city of Trondheim and the surrounding rural areas. About 700 patients were admitted every year.

Participants were patients consecutively admitted to the 2 emergency units in the hospital during the study period from October 2004 to December 2005 except three short breaks during vacations, totally 9 weeks.

Eligible participants were patients who on admittance fulfilled at least one of the following criteria: They were considered clinically to be under the influence of either substance(s), benzodiazepine(s) not used as prescribed by a physician, or the patient`s substance use was considered to affect the actual admission. Also patients initially answering negatively regarding substance use were included if they tested positively for one or more substances in an on-site urine drug of abuse screening test and they then confirmed such substance use. Finally patients not fulfilling the above criteria but who had received treatment for substance use the last 2 years prior to admission were included. This inclusion procedure was conducted to allow a possibility to intervene before discharge as this group of patients often has a short duration of stay. The final diagnoses according to ICD-10 were set at discharge with maximal information about the patients during the stay, i.e. after inclusion, randomisation and intervention. Thus, some of the participants may have been included in the study without fulfilling the ICD-10 criteria for disorders due to psychoactive substance use.

Patients were excluded if they were mentally retarded, suffering from dementia, serious brain damage or other conditions where adequate verbal communication was impossible. Patients were also excluded if they spoke neither Norwegian nor English. Finally, patients evaluated to be too seriously affected by their psychiatric disorder (e.g. severe psychosis symptoms) were excluded. The senior consultants at the department considered the inclusion and exclusion criteria for each patient.

### Procedure and assessments

At admittance all patients were examined by a physician. General clinical-chemical blood tests and an on-site urine screening-test for drug use (AccuSign DOA 5, Princeton Bio-Meditech Corporation, Princeton, NJ) were routine procedures at the department. AccuSign DOA 5 is an immunoassay urine test with acceptable reliability [26] for the qualitative detection of amphetamine, benzodiazepines, cannabis, cocaine and opiates and/or their metabolites. Also, all patients were assessed with Global Assessment of Functioning Scale [27], Split Version [28] at admittance.

In addition to clinical history, the patients were asked to fill in a self-report questionnaire. This questionnaire was used for baseline and follow-up assessments and described the frequency of alcohol and substance use and use of benzodiazepines not used as prescribed from a physician. The frequency as a measure of substance use severity was considered an adequate measure in this population with excessive substance use according to the eligible criteria. The frequency of substance use also will be related to function in general. The questionnaire has not been assessed for validity or reliability.

For the last three months prior to admission the respondents were asked to categorize their use of alcohol, cannabinoids, amphetamine, opiates, benzodiazepines, ecstasy and other substances respectively into 7 possible categories: never, monthly or more seldom than monthly, 2–3 times monthly, once a week, 2–3 days a week, 4–6 days a week and every day. The seven categories were recoded to correspond to number of days per month: never (0 days), monthly or more seldom than monthly (1 day), 2–3 times monthly (2.5 days), once a week (4.3 days), 2–3 days a week (10.7 days), 4–6 days a week (21.4 days) and every day (30 days).

For patients fulfilling the eligible criteria, a full description of the study procedure was given orally and written to each participant before written informed consent was obtained prior to inclusion and randomisation.

We expected different effect-sizes of the intervention according to different psychiatric clinical characteristics of the patients. Thus the patients were stratified into 3 groups based on the information available at the time of inclusion: non-psychosis and substance use, psychosis and substance use or non-psychosis and use of benzodiazepines not as prescribed by a physician and no other substance use.

The patients were randomly assigned to either the intervention group or the control group. Randomisation was performed by a web-based system developed and administered by the Unit of Applied Clinical Research, Institute of Cancer Research and Molecular Medicine, Norwegian University of Science and Technology, Trondheim, Norway. This was a block randomisation, with the block size for all 3 strata set to 10 in each strata group. The randomisation logarithm was programmed in PHP with a My SQL database. The clinicians making the baseline assessments had no information regarding the block size used for randomisation.

### Intervention; motivational interviewing

The intervention consisted of 2 sessions manual guided motivational interviewing delivered individually to the patients by a trained therapist [11]. The manual was developed by two motivational interviewing trainers in cooperation with the first author of this manuscript. Each session was planned to last 45 minutes. Depending on the patients` length of stay in the hospital, the second session took place on another day or later the same day.

In the first session the patients` ambivalence to substance use was explored. Also the severity of the patients` substance use was considered. In the second session the patients` experiences of substance use and prior attempts to change were explored to build intrinsic motivation for change. Actual readiness for change in substance use patterns and commitment to a change plan were focused on. The intervention was delivered in a motivational interviewing style. If they wanted, patients received information about, and referral to available follow-up treatment programs for substance use. The interviewer offered a written summary from the 2 sessions to each patient.

### Treatment as usual

Treatment as usual was individualized according to the clinical condition of the patients during the stay and in accordance with general national and international medical standards. It would usually include detoxification, pharmacotherapy, and general psychotherapy. Also, treatment would be given for any coexisting non-substance-related disorder, including psychiatric disorders.

General information about the harmful effects of substances and suggestions regarding treatment for substance use, including possible referral to specialty substance use treatment institutions, would be given. Planning of discharge with referral to out-patient and primary community health care after discharge usually would be included.

### Training and supervision of physicians and psychologists

A total of 15 therapists at the hospital fulfilled a training programme consisting of workshops and videotaping of role play using the manual with individual feedback on motivational interviewing micro-skills before the study started. This feedback was also continued during the study period.

### Diagnoses

The patients’ psychiatric diagnoses were set at discharge from the department according to the ICD-10 Diagnostic criteria for research [29] in a consensus meeting by the department staff, including at least 2 senior consultants.

### Primary outcome: substance use

The primary outcome was days per month of substance use the last 3 months. The most frequently used substance was measured. In the follow-up, questionnaires were sent by mail to the patients 3, 6, 12 and 24 months after inclusion. As compensation for participation, the patients received a lottery ticket worth 3 GBP for each questionnaire returned. If we did not receive the questionnaire during the following 14 days, nurses from the department, blind to treatment allocation, made telephone calls to ask for patients` reply.

Based on the size of previous studies at the time of the study period [30,31], we planned to include 100 patients in each trial arm. Since the recruitment was slower than anticipated, we ended the inclusion period when 135 patients were included in total.

### Statistical methods

The descriptive characteristics of the sample were generated using SPSS for windows version 17. The estimation of the intervention effect was performed according to the intention-to-treat principle. A multilevel linear repeated measures regression model with random slopes was used for analyses in STATA 11 for windows (Stata Corp., College Station, TX). This approach uses all available information and is less susceptible to bias under the assumption of missing at random [32,33]. Each follow-up wave was added to the model as a dummy variable (i.e. 3 months, 6 months, 12 months, 24 months and the admittance registration as a reference). To assess differences between intervention and control during follow-up, interaction terms between the intervention and each registration time-point were included in the model. In an additional analysis we also tested a model which included those participants with complete registrations of substance use frequency on all follow-up time points. As an indicator of substance use clustering at the patient level, we estimated an *Intra class correlation coefficient* (ICC) [32]. As a sensitivity analysis, we adjusted for gender, age and the Global Assessment of Functioning Scale, [27] Split Version [28] score at admittance. Furthermore, we performed an analysis using frequency of alcohol use as the outcome, and an analysis where the outcome measure was calculated without the use of benzodiazepines. Finally, we analysed on the number of daily doses within 30 days, where daily doses is defined as the sum of the number of days of use for all substances combined in each individual.

## Results

### Participants

**Figure 1:**
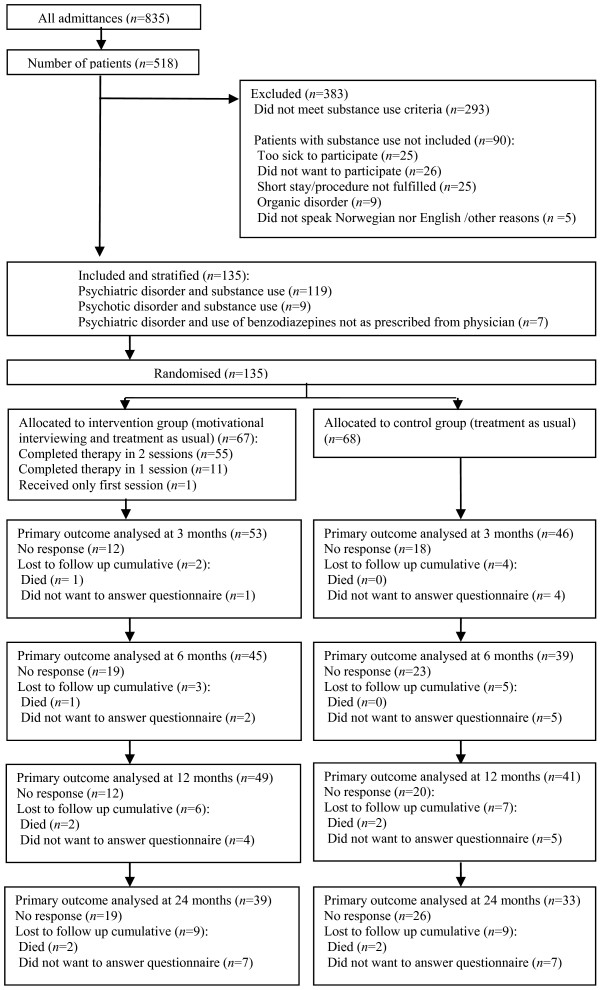
Recruitment and attrition according to CONSORT guidelines.

Recruitment and attrition according to CONSORT guidelines [34] are presented in Figure 1.

From a total of 835 admittances representing 518 patients, 293 patients had no known substance use. Of the 225 patients with substance use, 90 were excluded according to the exclusion criteria. Thus, a total of 135 patients were stratified and randomly allocated, 68 patients to the control group and 67 patients to the intervention group. A urine sample for on-site drugs of abuse screening-test was obtained from 122 of the 135 patients.

Baseline characteristics are presented in Table 1. The patients` mean age was 36.5 years (SD = 13.7), ranging from 18 to 80 years old. They were predominantly men, tended to be single and receiving disability payment and living in their own homes. According to Global Assessment of Functioning Scale, Split Version, the mean general functioning score of the patients was 43.1 (SD = 8.4, range 22–65), and the mean general symptom score were 41.4 (SD = 9.4, range 5 – 65).

**Table 1:** Baseline characteristics

	**Intervention group N (%)**	**Control group N (%)**	**p-value **^**a**^
Male	38 (56.7)	40 (58.8)	0.941
Single, separated or widow	52 (77.6)	52 (76.5)	1.000
Receiving disability payment, pensions or benefits	48 (71.6)	54 (79.4)	0.395
No housing, institution or hospice	8 (11.9)	14 (20.6)	0.260
Education			0.506
<10 years of schooling	20 (29.9)	20 (29.4)	
10-12 years of schooling	29 (43.3)	35 (51.5)	
>12 years of schooling	18 (26.9)	13 (19.1)	
Admitted by coercion	8 (11.9)	9 (13.2)	1.000
	**Mean (SD)**	**Mean (SD)**	**p-value **^**b**^
Age	36.9 (14.0)	36.1 (13.4)	0.728
Global Assessment of Functioning Scale, Split Version, Symptom score	41.9 (9.2)	40.9 (9.7)	0.517
Global Assessment of Functioning Scale Split Version, Function score	43.6 (8.4)	42.5 (8.4)	0.457

^a^ χ ^2^ –test.

^b^ independent –samples t-test.

### Substance use at baseline

Alcohol was the most frequently used substance, with 55 patients reporting use in both the intervention and the control group respectively (Table 2). Mean monthly days of use for most frequently used substance the last 3 months were 13.6 days (SD = 10.7) in the intervention group and 11.7 days (SD = 9.9) in the control group.

**Table 2:** Mean number of days of monthly substance use the last 3 months reported at baseline

	**Intervention group (*n*** **= 67)**			**Control group (*n*** **= 68)**		
	**Answered**^**a **^**N**	**Number of days, mean(SD)**	**Any use N (%)**	**Answered**^**a **^**N**	**Number of days, mean(SD)**	**Any use N (%)**
Alcohol	60	10.4 (9.4)	55 (91.7)	57	10.1 (8.3)	55 (96.5)
Amphetamine	67	1.4 (5.3)	11 (16.4)	66	1.0 (3.6)	15 (22.7)
Benzodiazepines	63	2.4 (5.6)	17 (27.0)	59	1.6 (5.6)	9 (15.3)
Cannabinoids	67	5.1 (9.7)	27 (40.3)	66	3.0 (7.3)	18 (27.2)
Ecstasy	66	0.0 (0.4)	0 (0.0)	64	0.1 (0.4)	4 (6.3)
Opioids	66	1.7 (6.3)	7 (10.6)	64	1.1 (5.3)	8 (12.5)
Main substance^b^	67	13.6 (10.7)		66	11.7 (9.9)	

^a^ Number of patients answering the questionnaire about the actual substance.

^b^ The substance most frequently used.

### Psychiatric diagnoses

The primary diagnoses at discharge were substance use disorders in 58 (43.0%) patients, affective disorders in 46 (34.1%), psychotic disorders in 8 (5.9%) and other disorders in 23 (17.0%) patients respectively. Of the 135 patients, a total of 117 (86.7%) patients had at least one substance use diagnosis. Among the substance use diagnoses, 6 (5.1%) patients had psychotic disorder due to psychoactive substance use, 61 (52.1%) had dependence syndrome, 46 (39.3%) harmful use and 4 (3.4%) acute intoxication. The median days of hospitalization for the intervention group was 6 (range 0–854, Q1: 3, Q3: 20), and for the control group 5 (range 0–886, Q1: 3, Q3: 10) respectively. Mann Whitney showed no statistical significant difference in days of hospitalization between the intervention group and the control group (p = 0.5).

### Intervention

Of the 67 patients allocated to motivational interviewing, 55 (82.0%) completed both sessions separately. 11 (16.4%) patients received the first and the second session continuously with no time in between. One patient did not want the second session. Some patients did not want a written summary. Of the 55 patients completing both sessions 2 received the second session as out-patients after discharge. Median days from admittance to the first intervention were 2.0 (range 0–35) and mean duration of the total interventions was 82.8 minutes (SD = 26.5) with a minimum of 35 and a maximum of 150 minutes.

By accident, one patient was included and given intervention twice, with 90 days between interventions. We used the patients` first intervention and disregarded the other.

### Follow-up and attrition

Follow-up questionnaires were completed by 99 (73.3%) patients at 3 months, by 84 (62.2%) at 6 months, by 90 (66.7%) at 12 months and by 72 (53.3%) at 24 months (Figure 1). Response during follow-up was assessed with a random intercept logistic regression model. We found lower response during follow up for the control group compared with the intervention group (odds ratio 0.5, 95% CI 0.3 to 1.0). Responders and non-responders at 24 months follow-up, were compared regarding baseline substance use, gender, age, housing, marital status, educational level and Global Assessment of Functioning scale, Split version, with no statistical significant differences between the groups, other than a difference in income, showing a higher proportion of the responders having work or student loans as an income, than the non-responders (p = 0.02).

### Primary outcome

**Figure 2:**
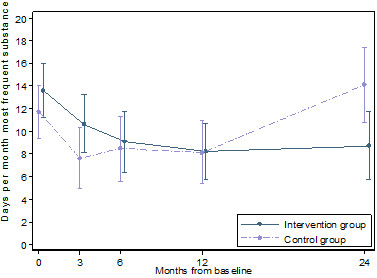
**Estimated days per month of substance use the last 3 months.** With 95% confidence intervals (vertical lines) as a function of months from baseline. Estimates based on a linear mixed model.

There was considerable clustering of substance use at the patient level as indicated with an ICC of 0.25. Both the intervention and the control groups showed similar reductions in days of substance use the first 12 months (Figure 2 and Table 3). At 12 months the control group had reduced their average substance use with 3.6 days (95% CI 0.3 to 6.9), while the intervention group had reduced their substance use with 5.4 days (95% CI 2.4 to 8.5) - a net difference in reduction between the groups of 1.8 days (95% CI −2.7 to 6.3). At 2 year follow-up, the control group had increased their substance use with 2.4 days (95% CI −1.5 to 6.3), whereas the intervention group had reduced their monthly substance use with 4.9 days (95% CI 1.2 to 8.6) compared to baseline. The net difference from baseline to 2 year follow-up was 7.3 days (95% CI 1.9 to 12.6, p < 0.01) in favour of the intervention group. In additional analysis we adjusted for gender, age and baseline Global Assessment of Functioning Scale Split Version, but this did not substantially change the results.

**Table 3:** Difference in substance use the last 3 months according to time and intervention

	**β**^**a**^	**95% CI**	**p-value**
Intervention compared with control at start of treatment	1.88	−1.50 to 5.22	0.271
Time 3 months compared with start of treatment ^b^	−4.13	−7.17 to −1.10	0.008
Time 6 months compared with start of treatment ^b^	−3.24	−6.46 to −0.02	0.049
Time 12 months compared with start of treatment ^b^	−3.59	−6.85 to −0.34	0.030
Time 24 months compared with start of treatment ^b^	2.37	−1.54 to 6.28	0.235
Time 3 months ^a^ Intervention ^c^	1.18	−3.00 to 5.35	0.580
Time 6 months ^a^ Intervention ^c^	−1.28	−5.71 to 3.15	0.571
Time 12 months ^a^ Intervention ^c^	−1.81	−6.27 to 2.65	0.425
Time 24 months ^a^ Intervention ^c^	−7.26	−12.64 to −1.89	0.008
Constant	11.72		

Estimated days per month with 95% confidence intervals, using a linear mixed model.

^a^ Unstandardized regression coefficient.

^b^ Estimate for the control group.

^c^ Estimate for additional effect of time for the intervention group compared with the control group relative to start of treatment.

An analysis comprising only alcohol use showed similar results as the main analysis (see Additional file 1: Table S1). Both groups reduced number of days using alcohol in the first year. At 12 months the control group had reduced their average alcohol use with 3.7 days (95% CI 1.0 to 6.4), while the intervention group had reduced their alcohol use with 4.0 days (95% CI 1.5 to 6.4). At 24 months of follow-up, the control group had increased their alcohol use with 0.9 days monthly (95% CI −2.2 to 4.1) compared to baseline, while the intervention group had reduced their alcohol use with 3.8 days per month (95% CI 0.9 to 6.7) in the same period. There was a net difference in use of alcohol from baseline to 24 months between the intervention and the control group of 4.7 days per month (95% CI 0.4 to 9.0).

A model without self-reported use of benzodiazepines, showed similar results as reported in Figure 2 (see Additional file 2: Table S2). Both the intervention and the control group reduced the number of monthly days with substance use the last 3 months the first year. After 12 months the control group showed a reduction of 4.5 days (95% CI 1.7 to 7.3), while the intervention group had reduced their substance use with 6.2 days (95% CI 3.6 to 8.8). At 24 months of follow-up the control group had decreased their use by 0.5 days (95% CI −2.8 to 3.7) compared with start of treatment, while the intervention group showed a reduction of 6.2 days (95% CI 3.2 to 9.3).

Analysing the number of daily doses including all substances each patient used, not only the main substance for each patient, showed similar results (see Additional file 3: Table S3). Both groups reduced substance use the first 12 months, while the control group was close to the baseline substance use at 24 months – an increase of 0.2 days (95% CI −5.5 to 6.0) compared with baseline. The intervention group reduced the number of daily doses of substances per month with 9.4 daily doses (95% CI 3.9 to 14.9) from baseline to 24 months. Excluding benzodiazepines from the analysis did not alter these results.

A complete case analysis showed similar results as reported in Figure 2 (see Additional file 4: Table S4), but the precision of the estimates was considerably reduced. Compared with baseline the control group had 0.7 more days (95% CI −5.0 to 6.5) with substance use at 24 months of follow-up, while the intervention group had reduced their number of days with substance use with 2.4 days (95% CI −2.6 to 7.5). Relative to baseline, the net difference was 3.1 days (95% CI −4.5 to 10.8) more of drug use last month in the control arm than the intervention arm.

## Discussion

The present study suggests that motivational interviewing combined with treatment as usual to adult patients with comorbid substance use admitted to a psychiatric emergency unit, may give a sustained reduction in patients’ frequency of substance use at 24 months follow-up compared to treatment as usual only. The reduction the first 12 months of follow-up was similar in the intervention group and the control group. However, at 24 months the control group had an increase in frequency of substance use back to the level at admittance while the intervention group seemed to manage to stay away from substance use at a significantly higher degree.

A recent Cochrane review finds an early effect of motivational interviewing compared to no treatment, reporting the effect to be strongest post-intervention and weaker at short and intermediate follow-up. For long term follow-up the effect was not significant. Compared to treatment as usual there was no effect in favour of motivational interviewing [13]. This is in line with the current study finding no effect of motivational interviewing added to treatment as usual the first 12 months. However, this study suggests an effect of motivational interviewing added to treatment as usual on a long term follow-up. Actually, the findings of the current study are in accordance with a 3 year follow-up randomised controlled study where brief intervention (30 minutes) in motivational interviewing style and treatment as usual compared to treatment as usual only was delivered to patients with alcohol disorders admitted to a trauma centre. In that study at 3 year follow-up patients in the intervention group were less likely to be arrested for driving under the influence than the control group [35]. In a randomised controlled study on heavy drinkers among college students effects were measured annually for 4 years, showing that the intervention group receiving brief motivational interviewing reported less drinking and less negative consequences of drinking also after 4 years [36]. To our knowledge, randomised controlled trials with long term follow-up on the effect of a short intervention with motivational interviewing and treatment as usual compared to treatment as usual are sparse [13,15,37].

The present study indicates that motivational interviewing added to treatment as usual may lead to sustained protection against substance use relapses in the intervention group compared to the control group at 2 year follow-up. As motivational interviewing may induce lasting intrinsic change in the person it is interesting to look broader into psychotherapy research on change. In line with the current study, it has been shown that insight at the end of short-term dynamic therapy may have a sustained effect on patients’ change of behavior at 2 years post treatment [16].

In the present study, there was a reduction in substance use in both the intervention group and the control group during the first 12 months after inclusion, with no substantial difference between the two groups. One possible explanation for this finding may be that the patients acutely admitted to a psychiatric emergency unit are in crisis and have higher levels of substance use at admission to the hospital than they would usually have. Thus, regression to the mean may explain some of the reductions in primary outcome during the first 12 months of follow-up. A second explanation may be linked to the effects of treatment as usual, which might help recovery and thereby reduce the use of substances after discharge. A reduction in the use of alcohol has been found following treatment as usual in patients with psychotic disorders and alcohol use, typically when involving the use of alcohol assessments [24]. A third moment is that increased focus on substance use during the study period may have resulted in a treatment as usual at the hospital more appropriate for substance use problems. Also training and supervising therapists in motivational interviewing during the inclusion period may have caused a leakage of motivational interviewing therapist style to the treatment as usual group, masking the possible initial effects of the motivational interviewing intervention.

The current study has some limitations. One main limitation in interpreting the difference between the two groups becoming apparent at 24 months post intervention is the loss to follow-up. At 24 months the follow-up rate of responders was low (53.3%). There was also a higher response during follow-up in the intervention group, and loss to follow-up could possibly have affected the results and the number of patients included (135) may be too small to compensate for the mentioned loss to follow-up. It is also worth to notice that although the complete case analysis gave comparable results as our main analysis, the group difference was somewhat smaller than in the main analysis. However, to partially compensate for this, we applied a multilevel linear repeated measures regression model which uses all available information and is less susceptible to bias under the assumption of missing at random [32]. Loss to follow-up may reflect the clinical nature of patients with substance use admitted to a psychiatric emergency unit, and conducting studies in this field can be challenging though not less important since randomised controlled trials on these patients are still few [24,25]. There were no statistical significant differences regarding baseline characteristics (Table 1) between the intervention and the control group. Looking at baseline characteristics of responders and non-responders (at 24 months follow-up), indicated that having a steady income was associated with higher response rate. This, however, was similar for both the intervention and the control group and thus is not expected to have influenced the result.

A second limitation is that the interventions were not videotaped or recorded. However, they were manual-guided. Also regular video feedback was arranged to validate the counsellors` motivational interviewing style.

Finally, the self-report questionnaires have not been assessed for validity or reliability, and self-report questionnaire only at follow-up is a limitation and it might have improved the study if biochemical analyses at follow-up had been performed.

However, several strengths may be mentioned. The study was a randomised controlled trial performed in a naturalistic setting representing the daily clinical situation in a psychiatric emergency unit. The patients were from both urban and rural areas and the study was conducted in a single hospital, giving minimal variation in treatment as usual. The patients were mainly men and single. Alcohol was the most frequently used substance. This resembles studies from other countries [2,38]. To compensate for the limitations of self-reporting, urine screening analyses were performed at baseline.

The patients` psychiatric diagnoses were set at discharge from the department according to ICD-10 Diagnostic criteria for research in a consensus meeting with at least 2 senior consultants. Thus the diagnoses were not confirmed at admittance and patients not fulfilling substance use diagnoses at admittance were also eligible for inclusion. However, 117 of the 135 included patients received at least one diagnosis of substance use disorder at discharge, indicating the severity of the substance use problems for these patients.

## Conclusions

The present study suggests that two sessions with motivational interviewing in addition to treatment as usual to patients with comorbid substance use admitted to a psychiatric emergency unit may well give substantial reduction in days of substance use at 24 months follow-up compared to the group receiving treatment as usual. The limited duration of stay and the little time available for intervention are challenges in a psychiatric emergency unit. This makes short interventions like motivational interviewing suitable. Both the intervention group and the control group showed substantial substance use reduction during the first 12 months. For the group receiving motivational interviewing in addition to treatment as usual however, the study indicates that the reduction may prevail also at 24 months of follow-up, which seems not to be the case for the group receiving treatment as usual only. Thus, the present study suggests sustained effects of a short intervention with motivational interviewing. Further randomised controlled studies in different psychiatric settings are needed. The studies should have a long term follow-up beyond 12 months.

## Competing interests

The authors declare that they have no competing interests. The study was fully supported materially and financially by St. Olav University Hospital, Trondheim, Norway.

## Authors’ contributions

GB and GM contributed to the conception and design, the analysis and interpretation of the data. CØ, SKR, JHB and PR contributed to the analysis and interpretation of the data. All authors drafted the article and revised it critically for important intellectual content. All authors read and approved the version of the manuscript to be published.

## Pre-publication history

The pre-publication history for this paper can be accessed here:

http://www.biomedcentral.com/1471-244X/13/93/prepub

## Supplementary Material

Additional file 1: Table S1 Difference in substance use (excluding benzodiazepines) the last 3 months according to time and intervention. Estimated days per month with 95% confidence intervals, using a linear mixed model. Click here for file

Additional file 2: Table S2 Difference in substance use (excluding benzodiazepines) the last 3 months according to time and intervention. Estimated days per month with 95% confidence intervals, using a linear mixed model. Click here for file

Additional file 3: Table S3 Difference in daily doses including all substances the last 3 months according to time and intervention. Estimated days per month with 95% confidence intervals, using a linear mixed model. Click here for file

Additional file 4: Table S4 Difference in substance use the last 3 months according to time and intervention for cases with no missing observations (complete cases). Estimated days per month with 95% confidence intervals, using a linear mixed model. Click here for file
