# Supplementary material for: The effects of motivational interviewing on patients with comorbid substance use admitted to a psychiatric emergency unit - a randomised controlled trial with two year follow-up
Source: BMC Psychiatry. 2013 Mar 21;13:93. doi: 10.1186/1471-244X-13-93 (PMC3618135; doi:10.1186/1471-244X-13-93)
Supplement: Additional file 1: Table S1 — Difference in substance use (excluding benzodiazepines) the last 3 months according to time and intervention. Estimated days per month with 95% confidence intervals, using a linear mixed model. [file 1471-244X-13-93-S1.docx]

**Table S1 Difference in alcohol use the last 3 months according to time and intervention.** Estimated days per month with 95% confidence intervals, using a linear mixed model

|  | β^a^ | 95% CI | p-value |
| --- | --- | --- | --- |
| Intervention compared with control at start of treatment | 0.35 | -2.53 to 3.23 | 0.812 |
| Time 3 months compared with start of treatment ^b^ | -3.02 | -5.53 to -0.50 | 0.019 |
| Time 6 months compared with start of treatment ^b^ | -3.53 | -6.18 to -0.87 | 0.009 |
| Time 12 months compared with start of treatment ^b^ | -3.70 | -6.35 to -1.04 | 0.006 |
| Time 24 months compared with start of treatment ^b^ | 0.91 | -2.22 to 4.05 | 0.569 |
| Time 3 months ^a^ Intervention ^c^ | -0.12 | -3.55 to 3.30 | 0.944 |
| Time 6 months ^a^ Intervention ^c^ | -0.11 | -3.73 to 3.50 | 0.951 |
| Time 12 months ^a^ Intervention ^c^ | -0.28 | -3.90 to 3.33 | 0.879 |
| Time 24 months ^a^ Intervention ^c^ | -4.71 | -8.99 to -0.42 | 0.031 |
| Constant | 9.68 |  |  |

^a^ Unstandardized regression coefficient

^b^ Estimate for the control group

^c^ Estimate for additional effect of time for the intervention group compared with the control group relative to start of treatment
