# Supplementary material for: The effects of motivational interviewing on patients with comorbid substance use admitted to a psychiatric emergency unit - a randomised controlled trial with two year follow-up
Source: BMC Psychiatry. 2013 Mar 21;13:93. doi: 10.1186/1471-244X-13-93 (PMC3618135; doi:10.1186/1471-244X-13-93)
Supplement: Additional file 2: Table S2 — Difference in substance use (excluding benzodiazepines) the last 3 months according to time and intervention. Estimated days per month with 95% confidence intervals, using a linear mixed model. [file 1471-244X-13-93-S2.docx]

**Table S2 Difference in substance use (excluding benzodiazepines**) **the last 3 months according to time and intervention.** Estimated days per month with 95% confidence intervals, using a linear mixed model

|  | β^a^ | 95% CI | p-value |
| --- | --- | --- | --- |
| Intervention compared with control at start of treatment | 2.12 | -0.96 to 5.20 | 0.177 |
| Time 3 months compared with start of treatment ^b^ | -3.73 | -6.35 to -1.11 | 0.005 |
| Time 6 months compared with start of treatment ^b^ | -4.36 | -7.14 to -1.58 | 0.002 |
| Time 12 months compared with start of treatment ^b^ | -4.47 | -7.26 to -1.69 | 0.002 |
| Time 24 months compared with start of treatment ^b^ | -0.48 | -3.73 to 2.76 | 0.770 |
| Time 3 months ^a^ Intervention ^c^ | -0.34 | -3.94 to 3.26 | 0.854 |
| Time 6 months ^a^ Intervention ^c^ | -1.10 | -4.92 to 2.72 | 0.571 |
| Time 12 months ^a^ Intervention ^c^ | -1.74 | -5.55 to 2.07 | 0.370 |
| Time 24 months ^a^ Intervention ^c^ | -5.72 | -10.18 to -1.27 | 0.012 |
| Constant | 11.15 |  |  |

^a^ Unstandardized regression coefficient

^b^ Estimate for the control group

^c^ Estimate for additional effect of time for the intervention group compared with the control group relative to start of treatment
