# Supplementary material for: The effects of motivational interviewing on patients with comorbid substance use admitted to a psychiatric emergency unit - a randomised controlled trial with two year follow-up
Source: BMC Psychiatry. 2013 Mar 21;13:93. doi: 10.1186/1471-244X-13-93 (PMC3618135; doi:10.1186/1471-244X-13-93)
Supplement: Additional file 3: Table S3 — Difference in daily doses including all substances the last 3 months according to time and intervention. Estimated days per month with 95% confidence intervals, using a linear mixed model. [file 1471-244X-13-93-S3.docx]

**Table S3 Difference in daily doses including all substances** **the last 3 months according to time and intervention.** Estimated days per month with 95% confidence intervals, using a linear mixed model

|  | β^a^ | 95% CI | p-value |
| --- | --- | --- | --- |
| Intervention compared with control at start of treatment | 4.29 | -2.39 to 10.97 | 0.208 |
| Time 3 months compared with start of treatment ^b^ | -5.22 | -10.45 to 0.02 | 0.051 |
| Time 6 months compared with start of treatment ^b^ | -5.02 | -10.42 to 0.38 | 0.069 |
| Time 12 months compared with start of treatment ^b^ | -6.30 | -11.64 to -0.95 | 0.021 |
| Time 24 months compared with start of treatment ^b^ | 0.24 | -5.51 to 5.98 | 0.936 |
| Time 3 months ^a^ Intervention ^c^ | -0.21 | -7.31 to 6.89 | 0.953 |
| Time 6 months ^a^ Intervention ^c^ | -2.68 | -10.05 to 4.69 | 0.476 |
| Time 12 months ^a^ Intervention ^c^ | -0.72 | -7.98 to 6.53 | 0.845 |
| Time 24 months ^a^ Intervention ^c^ | -9.63 | -17.58 to -1.67 | 0.018 |
| Constant | 15.80 |  |  |

^a^ Unstandardized regression coefficient

^b^ Estimate for the control group

^c^ Estimate for additional effect of time for the intervention group compared with the control group relative to start of treatment
