# Supplementary material for: The effects of motivational interviewing on patients with comorbid substance use admitted to a psychiatric emergency unit - a randomised controlled trial with two year follow-up
Source: BMC Psychiatry. 2013 Mar 21;13:93. doi: 10.1186/1471-244X-13-93 (PMC3618135; doi:10.1186/1471-244X-13-93)
Supplement: Additional file 4: Table S4 — Difference in substance use the last 3 months according to time and intervention for cases with no missing observations (complete cases). Estimated days per month with 95% confidence intervals, using a linear mixed model. [file 1471-244X-13-93-S4.docx]

**Table S4 Difference in substance use the last 3 months according to time and intervention for cases with no missing observations (complete cases).** Estimated days per month with 95% confidence intervals, using a linear mixed model

|  | β^a^ | 95% CI | p-value |
| --- | --- | --- | --- |
| Intervention compared with control at start of treatment | -2.22 | -7.87 to 3.43 | 0.441 |
| Time 3 months compared with start of treatment ^b^ | -5.06 | -10.15 to 0.04 | 0.052 |
| Time 6 months compared with start of treatment ^b^ | -4.83 | -9.96 to 0.30 | 0.065 |
| Time 12 months compared with start of treatment ^b^ | -5.23 | -10.78 to -0.27 | 0.039 |
| Time 24 months compared with start of treatment ^b^ | 0.73 | -5.02 to 6.47 | 0.805 |
| Time 3 months ^a^ Intervention ^c^ | 4.49 | -2.28 to 11.27 | 0.194 |
| Time 6 months ^a^ Intervention ^c^ | 2.92 | -3.89 to 9.74 | 0.400 |
| Time 12 months ^a^ Intervention ^c^ | -0.64 | -7.63 to 6.35 | 0.857 |
| Time 24 months ^a^ Intervention ^c^ | -3.14 | -10.78 to 4.50 | 0.420 |
| Constant | 14.18 |  |  |

^a^ Unstandardized regression coefficient

^b^ Estimate for the control group

^c^ Estimate for additional effect of time for the intervention group compared with the control group relative to start of treatment
